# Multi-Scale Tribo–Thermo–Viscoelastic Engineering of Sustainable Bio-Based Epoxy Through Hybrid Carbon Nano Architectures and Energy Partition Modeling

**DOI:** 10.3390/polym18060752

**Published:** 2026-03-19

**Authors:** Kiran Keshyagol, Pavan Hiremath, Rakesh Sharma, Muralishwara K, Santhosh K, Suhas Kowshik, Nithesh Naik

**Affiliations:** Manipal Institute of Technology, Manipal Academy of Higher Education, Manipal 576104, India

**Keywords:** sustainable bio-based epoxy, hybrid carbon nanofillers, wear resistance, dry sliding tribology, energy-efficient materials, SDG 9, SDG 12

## Abstract

This study investigates the multi-scale tribo–thermo–viscoelastic performance of a sustainable bio-based FormuLITE epoxy reinforced with single and hybrid carbon nanofillers (0.1 wt.% total loading) under dry sliding up to 50 N. Pin-on-disk tests at 10, 30, and 50 N showed a consistent reduction in contact pressure and wear volume in the order: neat epoxy > 0.1 CNT > 0.1 GNP > 0.1 ND > 0.1 CNT/GNP > 0.1 CNT/ND > 0.1 GNP/ND. At 50 N and 1500 m sliding distance, neat epoxy exhibited a wear volume of 13.43 mm^3^ and contact pressure of 13.4 N/cm^2^, while the GNP/ND hybrid reduced wear to 4.86 mm^3^ and contact pressure to 6.2 N/cm^2^, corresponding to reductions of 64% and 54%, respectively. The accelerating wear coefficient decreased from 2.9 × 10^−6^ to 8.5 × 10^−7^, confirming slower damage accumulation in hybrid systems. Time-dependent contact pressure analysis revealed reduced asymptotic intensity and suppressed mid-cycle pressure spikes, indicating enhanced tribolayer stability. Effective surface hardness increased from 0.18 GPa (neat epoxy) to 0.30 GPa (GNP/ND), while normalized wear decreased from 1.00 to 0.36. Enhanced damping behavior and improved thermal conductivity in hybrid systems promoted stress redistribution and minimized flash-temperature localization. An interfacial energy-partition framework calibrated to experimental wear data quantitatively linked effective driving pressure, tribofilm stabilization, and surface hardness to material removal. The results demonstrate that wear mitigation in sustainable bio-epoxy systems is governed by coupled mechanical, viscoelastic, and thermal energy redistribution, with GNP/ND hybrids providing the most stable tribological interface under severe sliding. The findings contribute to the development of durable and sustainable bio-epoxy composite systems for engineering applications, supporting broader goals of responsible material utilization and sustainable industrial innovation aligned with the United Nations Sustainable Development Goals (SDG 9 and SDG 12).

## 1. Introduction

Polymer-based tribological systems are increasingly replacing metallic components in automotive, aerospace, biomedical, and structural applications due to their low density, corrosion resistance, design flexibility, and energy efficiency [1]. However, under dry sliding conditions and elevated contact stresses, thermoset polymers remain susceptible to rapid material degradation through abrasive plowing, adhesive micro-junction formation, fatigue cracking, and thermal softening [2,3]. These degradation mechanisms become particularly severe under high-load regimes where localized stress concentration and flash-temperature rise accelerate bond scission and debris generation [4]. Improving wear resistance without compromising sustainability remains a critical challenge in advanced polymer engineering.

Bio-based epoxy systems derived from renewable feedstocks have emerged as promising alternatives to petroleum-derived matrices, offering reduced carbon footprint and improved environmental compatibility [5,6]. Nevertheless, the intrinsic brittleness and limited thermal transport capability of epoxy networks can result in stress localization and unstable tribolayer formation during sliding [7]. The development of multifunctional nano-reinforcement strategies that simultaneously enhance load-bearing stiffness, energy dissipation capacity, and thermal conductivity is therefore essential for advancing sustainable high-performance tribological materials. Such developments directly support Sustainable Development Goal (SDG) 12: Responsible Consumption and Production, through the adoption of bio-derived materials and enhanced durability, and SDG 13: Climate Action, by reducing lifecycle energy demand and material waste through extended component life.

Carbon-based nanofillers have attracted significant attention due to their exceptional mechanical and thermal properties. Multi-walled carbon nanotubes (MWCNTs) provide high aspect-ratio reinforcement and crack-bridging capability; graphite nanoplatelets (GNPs) offer two-dimensional shear accommodation through interlayer sliding; and nanodiamonds (NDs) contribute ultra-hard load-bearing nodes and micro-polishing effects [8,9,10,11]. While numerous studies have investigated these fillers individually, the mechanistic understanding of how single and hybrid nano-architectures redistribute interfacial energy during sliding remains incomplete. In particular, the accelerating wear regime characterized by nonlinear growth in material loss after prolonged sliding has received comparatively limited quantitative attention despite its relevance to component failure [12,13,14].

Most prior investigations focus primarily on average wear rates or coefficient of friction, without resolving the coupled roles of contact pressure evolution, viscoelastic damping behavior, thermal transport, and atomic-scale bond rupture probability [15,16,17]. Yet wear is fundamentally an energy-partition phenomenon: frictional work input at the interface must be either elastically stored, viscously dissipated, thermally conducted, or irreversibly consumed in bond scission and debris formation. A unified framework integrating these contributions is necessary to transition from empirical wear ranking to predictive materials design. In addition to nanofiller reinforcement strategies, ionic liquid (IL)-based approaches have recently attracted attention for modifying epoxy networks and curing behavior in sustainable composite systems. Ionic liquids can function as curing agents, catalysts, or multifunctional additives capable of influencing crosslinking reactions, interfacial interactions, and filler dispersion within epoxy matrices [18,19]. Recent studies have demonstrated IL-assisted curing and matrix modification in natural fiber–reinforced bio-epoxy composites and in epoxy/CNT nanocomposites, where ILs improve mechanical performance, interfacial bonding, and electrical or tribological properties [20,21]. These developments highlight the growing role of IL chemistry in sustainable epoxy composite design. However, the present study focuses on an alternative pathway in which nanocarbon fillers are used to stabilize the tribological interface and reduce wear in bio-epoxy systems under dry sliding conditions.

In this context, the present study investigates a bio-based FormuLITE epoxy system reinforced with 0.1 wt.% single and hybrid combinations of MWCNT, GNP, and ND fillers. Dry sliding tests were conducted at 10, 30, and 50 N loads to systematically evaluate contact pressure evolution, accelerating wear kinetics, and surface damage morphology. Transmission electron microscopy was used to confirm nanofiller morphology and dispersion; dynamic mechanical analysis quantified viscoelastic damping behavior (Tan δ); thermal conductivity measurements assessed heat-dissipation capability; and scanning electron microscopy revealed load-dependent wear mechanisms.

Beyond experimental characterization, this work proposes and calibrates an interfacial energy partition model linking apparent contact pressure, surface hardness, tribofilm stabilization, and wear response. The model is calibrated against experimentally measured wear volumes at extended sliding distance (1500 m) and correlated with time-dependent contact pressure asymptotics and SEM morphology. Furthermore, a unified tribo–thermo–viscoelastic scaling perspective is introduced to demonstrate how mechanical severity, internal damping, and thermal transport collectively govern wear mitigation.

The novelty of this work lies in: (i) the quantitative evaluation of the accelerating wear regime under high-load dry sliding; (ii) direct coupling of contact pressure evolution with viscoelastic and thermal parameters; (iii) interfacial mechanistic interpretation of single versus hybrid nanofiller synergy; and (iv) establishment of a calibrated energy-redistribution framework for mechanistic tribological interpretation and future predictive design of sustainable bio-epoxy systems. By integrating experimental tribology with multi-physics energy analysis, the study advances a scientifically grounded pathway for designing durable, environmentally responsible polymer composites capable of operating under severe mechanical conditions, thereby contributing to material longevity, resource efficiency, and sustainable manufacturing practices.

## 2. Background Study

Velmurugan et al. [22] investigated NaOH-treated Curauá/Areca fiber epoxy composites reinforced with Nano-SiO_2_ and reported a ~25% increase in tensile strength and ~20% improvement in flexural strength compared to untreated systems. Under optimized conditions, the specific wear rate decreased by approximately 30–35%, confirming improved tribological stability. Jagadeesh et al. [23] fabricated 30 wt% basalt micro-filler reinforced bio-based polypropylene (PP) and HDPE composites and reported that basalt addition reduced wear rate by ~40% and lowered friction coefficients under dry sliding compared to neat PP/HDPE. They found enhanced load bearing and improved interfacial adhesion contributed to these tribological improvements in sustainable thermoplastic composites. Sahu et al. [24] investigated HDPE hybrid polymer nanocomposites reinforced with multidimensional carbon fillers (ND, MWCNT, GNP) and showed that 0.1 wt% GNP/ND filler exhibited the lowest contact pressure with a corresponding ~30–40% reduction in wear volume compared with neat HDPE, linking wear performance directly to contact pressure and damping behavior (Tan δ ~ 0.5).

Pulikkalparambil et al. [25] surveyed sliding wear in sustainable biocomposites and reported that biofiller and synthetic filler additions can reduce wear rates by up to ~40–50% and lower friction under optimized sliding conditions, highlighting filler type and dispersion as key performance drivers. Paszkiewicz et al. [26] synthesized bio-based PPF-b-F-PTMO nanocomposites with 1D (CNFs, HNTs) and 2D (GNPs, organoclay) fillers and found that nanoparticle addition increased crystallinity by ~10–18% and tensile modulus by ~15–22% compared to neat polymer. Naik et al. [5] used a Taguchi and ANN coupled approach to study nanoclay (1–3 wt%) filled amine-cured bio-based epoxy and demonstrated that optimal nanoclay content reduced wear rate to ~0.0062 mg/min and frictional force to ~22.99 N, with nanoclay wt% contributing >90% influence on tribo-performance. Singh et al. [6] reviewed sustainable bio-based smart and intelligent composites and reported that bio-epoxy systems reinforced with natural fibers and nanocellulose exhibited up to ~30–45% improvements in mechanical/thermal performance and multifunctionality (e.g., sensing, actuation) compared to neat bio-matrices, emphasizing fiber/nanofiller integration and multifunctional design as key drivers in advanced sustainable composite development.

Sharma et al. [7] reviewed bio-based polyamide nanocomposites with nanoclay, carbon nanotubes, and graphene, reporting that incorporation of <5 wt% carbon-based nanofillers can enhance tensile strength by ~15–35% and electrical/thermal performance by up to ~2–3× compared to neat polyamide systems due to improved matrix–filler interaction and nanofiller dispersion. Kumar et al. [27] reported that incorporating 20–30 wt% agro-waste fillers into polymers reduced wear rate by ~35–50% and increased hardness by ~20–30% compared to neat matrices, emphasizing the role of filler dispersion and interfacial bonding in tribological improvement. Gupta et al. [28] reported that adding 20 wt% HAp and 3 wt% Al_2_O_3_ to PLA/CS bio-nanocomposites increased compressive strength by ~2.5× and modulus by ~1.7× while significantly reducing wear rate under optimized sliding conditions due to improved filler dispersion and load transfer. Faggio et al. [29] demonstrated that incorporating 5 phr CNT into bio-based BOMF/IPD epoxy increased electrical conductivity by ~8 orders of magnitude (10^−14^ to 10^−6^ S/m) and enabled stable Joule heating up to ~34 °C at 20 V in breathable cotton-based smart textiles.

Bhanderi et al. [30] present a comprehensive overview of eco-friendly polymer nanocomposites based on bio-based fillers, detailing their classification, synthesis routes, characterization methods, and wide-ranging applications. Dhanapalan et al. [31] reported that biosilica-modified banana fiber epoxy composites exhibited significantly reduced wear rate with increasing biosilica content, attributed to improved surface hardness and load-bearing capability under dry sliding conditions. Tadepalli [32] reported that incorporating 5 vol.% rice straw–derived carbon nanomaterial into 40 vol.% pineapple fiber/epoxy composite reduced the specific wear rate from 0.034 to 0.0067 mm^3^/nm (≈80.3% reduction) and lowered the COF from 0.38 to 0.21, demonstrating significant enhancement in tribological performance. Fragassa et al. [33] marine biocomposites experience significant tribological deterioration due to combined seawater exposure, moisture absorption, and biofouling, which accelerate fiber–matrix debonding and increase friction and wear.

These collective studies clearly demonstrate that filler engineering, interfacial optimization, and nano structural design significantly enhance tribological performance; however, a systematic understanding of time-dependent wear evolution and contact pressure governed damage progression in bio-based epoxy systems still remains limited, thereby motivating the present investigation.

## 3. Materials and Methodology

### 3.1. Materials and Composite Preparation

The composite specimens were prepared using a bio-based epoxy system comprising FormuLITE^®^ 2500A resin and FormuLITE^®^ 2401B hardener (Cardolite Specialty Chemicals, Karnataka, India), selected for its cardanol-based chemistry, good toughness, and favorable tribological performance; the properties can be found in authors’ previous work [34]. The resin and hardener were mixed according to the manufacturer-recommended stoichiometric ratio. Multi-walled carbon nanotubes (MWCNTs), graphite nanoplatelets (GNPs), and nanodiamonds (NDs) were used as nano-reinforcements. Transmission electron microscopy (TEM) analysis confirmed that the MWCNTs possessed outer diameters of approximately 8–25 nm with hollow concentric structures and micrometer-scale lengths, while GNP exhibited layered lamellar morphology with thicknesses typically in the range of 5–30 nm and lateral dimensions extending to a few hundred nanometers. Nanodiamonds were predominantly quasi-spherical to faceted particles with sizes between 4 and 8 nm, occasionally forming soft agglomerates due to their high surface energy. Single-filler and hybrid systems were fabricated with a total nanofiller loading of 0.1 wt.%, and hybrid compositions contained equal weight fractions of the respective fillers. To ensure homogeneous dispersion, the nanofillers were first incorporated into the epoxy resin using probe ultrasonication at 20 kHz under controlled cooling to avoid thermal effects, followed by high-shear mechanical stirring to break residual agglomerates. The mixture was subsequently vacuum-degassed, after which the hardener was introduced and gently mixed. The formulations were cast into molds and allowed to cure for 24 h at room temperature followed by post-curing at 80 °C for 3 h. Tribological specimens were cut from the composite laminates and stuck to cylindrical pins, resulting in non-uniform contact areas (~1.5–3.5 cm^2^). Prior to testing, the contact surfaces of the pins were lightly polished using fine abrasive paper to ensure a uniform and reproducible contact condition. The macro-scale geometry of the specimens was controlled to maintain consistent nominal contact area during testing.

### 3.2. Pin-on-Disk (POD) Tribometry

Dry sliding wear behavior was evaluated using a computer-controlled pin-on-disk tribometer against a polished stainless steel (SS316) counterface with surface roughness of approximately Ra ≈ 0.2 μm. Tests were conducted at normal loads of 10, 30, and 50 N, with a sliding speed of 1 m/s and a fixed sliding radius of 50 mm under ambient laboratory conditions. Wear depth was continuously monitored using an integrated LVDT sensor, and wear volume was calculated based on the measured displacement and contact geometry. Contact pressure was determined as the ratio of applied load to nominal contact area. Particular attention was given to identifying the run-in, steady-state, and accelerating wear phases from wear–distance curves. Each condition was repeated at least three times to ensure repeatability. The standard deviation of the repeated measurements was found to be within ±3–5% of the mean value, indicating good experimental repeatability.

### 3.3. Thermal Conductivity, Dynamic Mechanical Analysis (DMA) and Structural Examination

Thermal conductivity measurements were performed using a transient plane source technique on a Hot Disk TPS 2500 S analyzer (Hot Disk AB, Gothenburg, Sweden) with disk-shaped specimens, while dynamic mechanical analysis was carried out on a DMA Q800 instrument (TA Instruments, New Castle, DE, USA) in compression mode over a temperature range of 30–110 °C at a heating rate of 5 °C/min and a frequency of 1 Hz. Microstructural features and filler dispersion were examined using a JEOL JEM-2100 (JEOL Ltd., Tokyo, Japan) transmission electron microscope (TEM) operating at 200 kV, and the worn surfaces were further inspected using a Scanning Electron Microscope (SEM) to identify dominant wear mechanisms. All reported values represent mean results from repeated trials, and statistical scatter was considered in the analysis to ensure reliability of the measured trends.

## 4. Results and Discussion

### 4.1. Morphology and Dispersion of Nanofillers

**Figure 1:**
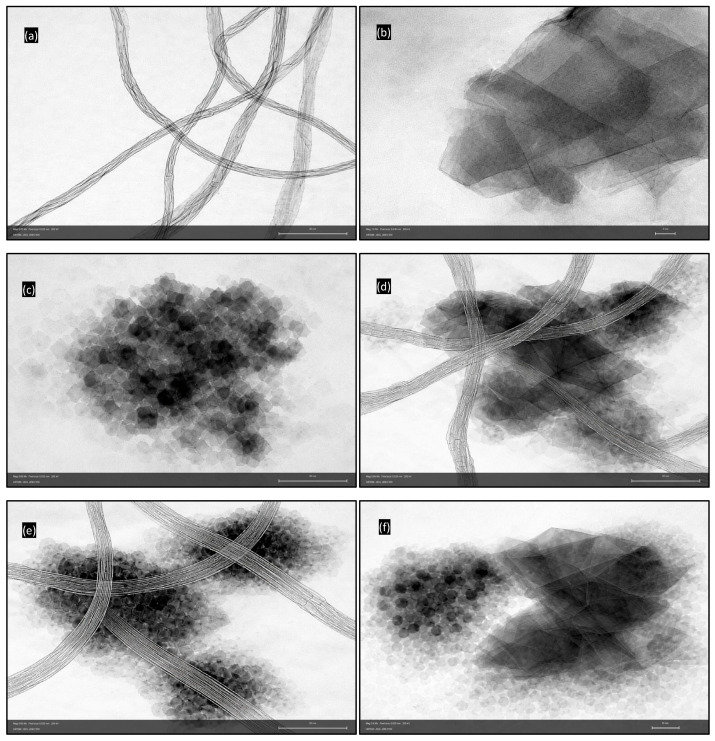
TEM images: (**a**) MWCNTs; (**b**) graphite nanoplatelets (GNPs); (**c**) nanodiamond (ND); (**d**) MWCNT/GNP hybrid; (**e**) MWCNT/ND hybrid; (**f**) GNP/ND hybrid.

**Figure 2:**
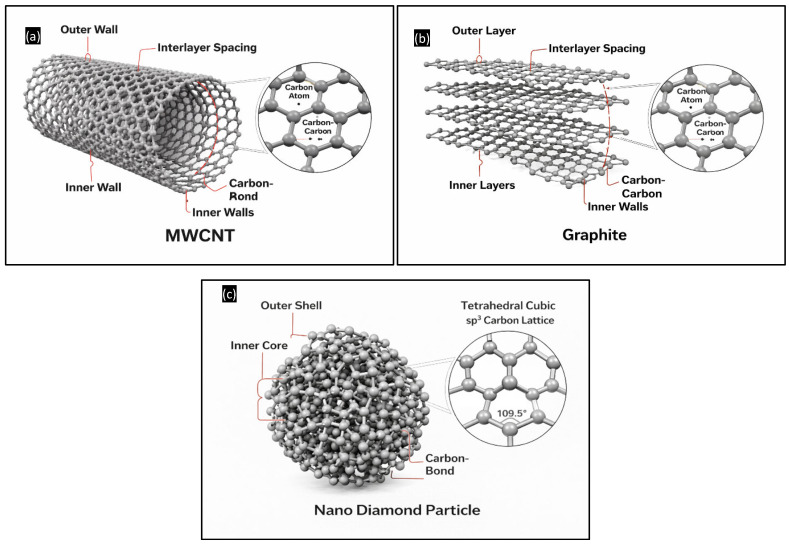
Hypothesized structural models: (**a**) MWCNT; (**b**) GNP layered; (**c**) nanodiamond tetrahedral lattice model.

The morphology and dispersion state of the nanofillers within the bio-based epoxy matrix were examined using transmission electron microscopy (TEM), since these parameters strongly influence interfacial bonding, load transfer efficiency, and tribological response. The TEM micrographs together with their hypothesized structural representations are presented in Figure 1a–f and Figure 2a–c.

The MWCNTs (Figure 1a) exhibit a characteristic multi-concentric tubular morphology with high aspect ratio and pronounced curvature. The nanotubes appear as entangled and partially aligned networks, forming a quasi-continuous reinforcing skeleton within the matrix. Such a structure promotes efficient stress transfer and crack-bridging during sliding. The integrity of the concentric walls indicates minimal structural damage during processing. The atomic-level architecture responsible for these properties is illustrated in the hypothesized MWCNT model (Figure 2a), where multiple graphene cylinders and interlayer spacing can be seen, explaining their high stiffness, resilience, and ability to sustain repeated contact stresses [35,36].

GNPs (Figure 1b) display a lamellar morphology composed of stacked graphitic layers with large lateral dimensions relative to thickness. The platelet-like geometry confirms their two-dimensional nature. These layered sheets enable interlayer sliding due to weak van der Waals forces, thereby providing intrinsic solid lubrication. Partial exfoliation and fairly uniform distribution are evident. The structural origin of this lubricity is clarified by the graphite model (Figure 2b), which shows stacked sp^2^-bonded carbon layers and interlayer spacing, directly correlating with their low shear resistance and tribofilm-forming capability [37,38].

Nanodiamond particles (Figure 1c) appear as near-spherical nanocrystallites in the few-nanometer range, occasionally forming small clusters. Their high electron contrast and faceted appearance are consistent with crystalline diamond. While some agglomeration is visible, the overall dispersion remains within the nanoscale regime. Their extreme hardness and modulus contribute to abrasion resistance and surface protection. The hypothesized nanodiamond structure (Figure 2c) depicts the tetrahedral sp^3^ carbon lattice with ~109.5° bond angles, explaining the exceptional hardness and elastic modulus associated with diamond-based reinforcements [39,40].

Hybrid nanofiller systems reveal synergistic dispersion and interaction mechanisms. In the MWCNT/GNP hybrid (Figure 1d), nanotubes bridge between graphite platelets, forming interconnected networks that combine load-bearing and lubricating effects. In the MWCNT/ND system (Figure 1e), nanodiamond clusters are observed along nanotube surfaces, indicating that CNTs serve as dispersion carriers that reduce ND agglomeration. The GNP/ND hybrid (Figure 1f) shows nanodiamond particles distributed around graphite layers, which can facilitate formation of durable and low-shear transfer films.

By correlating the TEM observations (Figure 1a–f) with the hypothesized structural models (Figure 2a–c), it becomes evident that each nanofiller contributes a distinct reinforcement mechanism: MWCNTs provide a one-dimensional load-transfer network, graphite offers two-dimensional lubricating layers, and nanodiamond introduces zero-dimensional ultra-hard reinforcement. This multiscale morphological synergy is expected to strongly influence contact pressure evolution, transfer film stability, and overall wear resistance during sliding.

### 4.2. Influence of Contact Pressure on Wear Performance in FormuLITE Epoxy System

**Figure 3:**
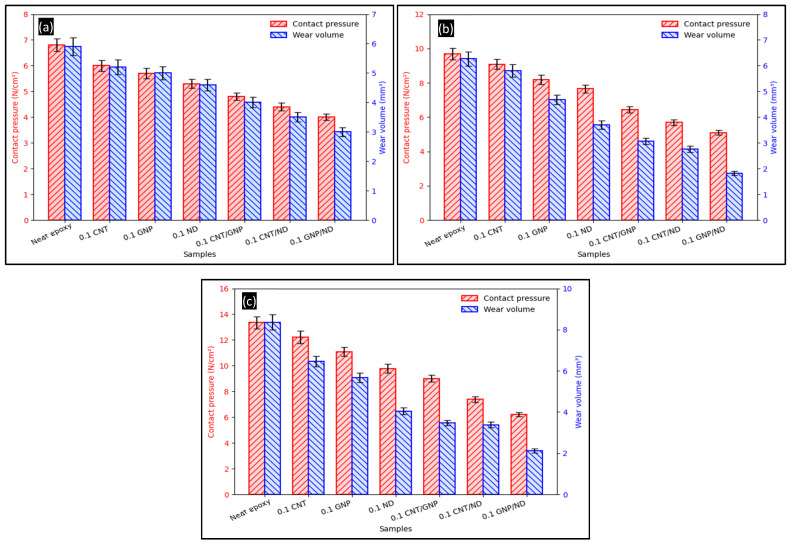
Contact pressure vs. wear volume from experimental wear–distance data at loads of (**a**) 10 N, (**b**) 30 N, and (**c**) 50 N.

The influence of contact pressure on the wear response of the FormuLITE epoxy system containing individual and hybrid nanofillers is summarized in Figure 3a–c for normal loads of 10, 30, and 50 N, respectively, where contact pressure and wear volume are plotted together for direct comparison. Table 1 shows the composition and formulation of samples. Under 10 N (Figure 3a), neat epoxy exhibits the highest contact pressure of ~6.8 N/cm^2^ with a corresponding wear volume of ~5.9 mm^3^, whereas progressive reductions are observed with nanofiller addition: 0.1 CNT (~6.0 N/cm^2^; ~5.2 mm^3^), 0.1 graphite (~5.7 N/cm^2^; ~5.0 mm^3^), and 0.1 ND (~5.3 N/cm^2^; ~4.6 mm^3^). Among the hybrids, the lowest values are consistently obtained for 0.1 GNP/ND (~4.0 N/cm^2^; ~3.0 mm^3^), followed by 0.1 CNT/ND (~4.4 N/cm^2^; ~3.6 mm^3^) and 0.1 CNT/GNP (~4.8 N/cm^2^; ~4.1 mm^3^). Relative to neat epoxy at 10 N, the GNP/ND hybrid reduces contact pressure by ~41% and wear volume by ~49%, confirming that the hybrid architecture is the most effective in mitigating both stress concentration and material removal at the sliding interface.

A similar ranking is retained at 30 N (Figure 3b). Neat epoxy shows ~9.8 N/cm^2^ and ~6.3 mm^3^, while 0.1 CNT (~9.1 N/cm^2^; ~5.7 mm^3^), 0.1 graphite (~8.2 N/cm^2^; ~4.7 mm^3^), and 0.1 ND (~7.7 N/cm^2^; ~3.7 mm^3^) display stepwise reductions. The hybrids again show the strongest improvement, with 0.1 GNP/ND reaching ~5.1 N/cm^2^ and ~1.9 mm^3^. Compared with neat epoxy at 30 N, GNP/ND lowers contact pressure by ~48% and wear volume by ~70%, indicating that the hybrid is especially effective once the interface enters a higher-stress regime where stable transfer film formation and shear-assisted sliding become critical.

At 50 N (Figure 3c), the absolute magnitudes rise as expected, but the comparative trend remains unchanged. At the final experimental point of the wear–distance curves shown in Figure 3, neat epoxy records ~13.4 N/cm^2^ and ~8.3 mm^3^, while 0.1 CNT (~12.3 N/cm^2^; ~6.5 mm^3^), 0.1 GNP (~11.1 N/cm^2^; ~5.7 mm^3^), and 0.1 ND (~9.9 N/cm^2^; ~4.1 mm^3^) show progressively lower wear. The hybrid GNP/ND combination again provides the minimum values (~6.2 N/cm^2^; ~2.1 mm^3^), corresponding to ~54% lower contact pressure and ~75% lower wear volume relative to neat epoxy at 50 N. Overall, across all three loads, the contact pressure and wear volume follow the consistent order of neat epoxy > 0.1 CNT > 0.1 GNP > 0.1 ND > 0.1 CNT/GNP > 0.1 CNT/ND > 0.1 GNP/ND, demonstrating that (i) adding a single nanofiller reduces interfacial stress and wear, and (ii) combining GNP with ND produces the largest synergistic benefit. Notably, the co-existence of graphite (easy interlayer shear) and nanodiamond (hard, load-bearing, micro-rolling/asperity polishing) plausibly promotes a more stable tribolayer and reduces localized plowing, thereby lowering both the contact pressure and the wear volume. Accordingly, the observed reduction in wear is interpreted in terms of improved load distribution and resistance to damage under the applied nominal contact condition, rather than as a direct measurement of real-time interfacial stress reduction.

In addition to wear volume measurements, the friction coefficient was continuously recorded during the pin-on-disk tests. The steady-state friction coefficient values for the different formulations are summarized in Table 2. The graphite-containing systems exhibit lower friction coefficients due to their lubricating nature, which contributes to improved tribological performance through the formation of stable transfer films at the sliding interface.

**Figure 4:**
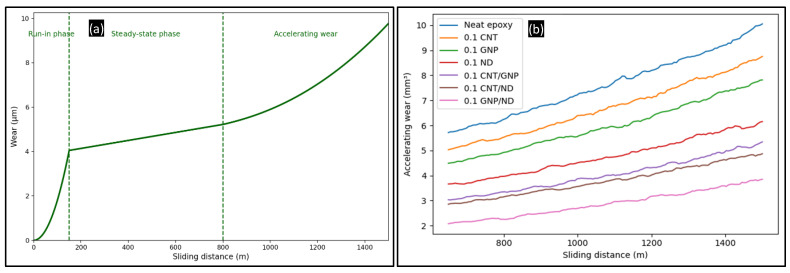
Wear output: (**a**) typical wear curve; (**b**) accelerating wear volume vs. sliding distance for all the samples at 50 N load.

The wear–distance response of the FormuLITE epoxy system follows a characteristic three-stage evolution as shown in Figure 4a. Stage I corresponds to the run-in phase, where rapid surface adaptation and asperity removal lead to an initially steep wear increase. Stage II represents a steady-state regime characterized by relatively stable wear progression and improved interfacial conformity. Stage III corresponds to the accelerating wear regime, manifested by a nonlinear rise in wear volume and associated with progressive surface degradation.

Although the dependence of wear on sliding distance and load has been widely reported, the accelerating wear regime remains less explored and is therefore emphasized here. Figure 4b presents the accelerating wear behavior under constant load conditions for all formulations. The onset of accelerating wear occurs at different sliding distances depending on filler type. For neat epoxy, the transition appears near ~750–800 m, whereas nanofiller-reinforced systems show delayed transitions. The delay is most pronounced for the GNP/ND hybrid, where acceleration initiates closer to ~1000–1100 m, demonstrating the beneficial role of hybrid reinforcement in stabilizing the tribo-surface.

The accelerating wear region was represented using quadratic polynomial fits, and the intercept values obtained from these fits provide a comparative measure of baseline wear magnitude. Higher intercepts correspond to greater inherent wear tendency, while lower intercepts indicate improved wear resistance. Among all formulations, the GNP/ND hybrid exhibits the lowest intercept and the slowest curvature growth, confirming its superior resistance to damage accumulation.

While contact pressure influences wear response, the stresses generated during sliding are primarily dissipated through micro-scale deformation, filler–matrix interactions, and debris formation rather than being retained as residual stresses. This dissipative mechanism contributes to the improved durability of nanofiller-reinforced systems. To support quantitative comparison and downstream modeling, quadratic polynomial fits for the accelerating regime are summarized in Table 3 using the form *W* = *aS*^2^ + *bS* + *c*, where *W* is wear volume (mm^3^) and *S* is sliding distance in m. The wear values discussed in Section 4.2 correspond to the final experimental data points obtained from Figure 3, whereas the values reported in Table 3 represent projected wear volumes calculated at a sliding distance of 1500 m using quadratic fitting.

### 4.3. Time-Dependent Contact Pressure Evolution and Asymptotic Intensity

**Figure 5:**
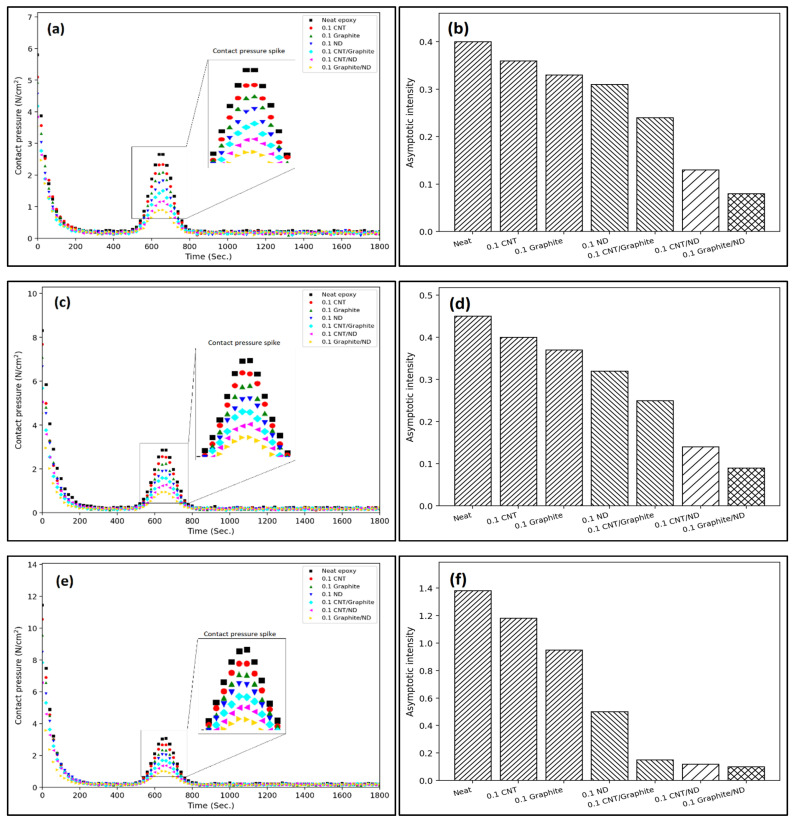
Contact pressure evolution and asymptotic intensity at 10, 30, and 50 N: (**a**,**c**,**e**) pressure vs. time; (**b**,**d**,**f**) asymptotic intensity.

The time-dependent evolution of contact pressure provides additional insight into the interfacial stability and load-sharing capability of the bio-epoxy nanocomposites during sliding. Figure 5a,c,e show the variation in contact pressure with time at applied loads of 10, 30, and 50 N, respectively. For all formulations, the contact pressure exhibits a characteristic decay–stabilization trend. An initially high contact pressure is observed at the beginning of sliding due to limited real contact area and asperity-dominated interaction. As sliding proceeds, surface conformity improves and a transfer layer begins to develop, resulting in a rapid reduction in contact pressure within the first ~100–200 s. This stage corresponds to the classical run-in regime.

Beyond the run-in period, the contact pressure approaches a quasi-steady level, indicating stabilization of the real contact area. Neat epoxy consistently maintains the highest contact pressure at all loads, reflecting its limited ability to redistribute stresses and protect the interface. In contrast, nanofiller-reinforced systems exhibit systematically lower pressure levels. Single-filler systems show moderate reductions, while hybrid systems demonstrate the most pronounced decreases. The GNP/ND hybrid consistently records the lowest contact pressure across the entire time span, confirming its superior load-sharing and interfacial stabilization capability.

Localized rise in contact pressure is observed around the mid-sliding interval (typically near 600–700 s). This transient increase is not indicative of catastrophic damage but is instead attributed to temporary tribolayer disruption or debris accumulation at the interface. Such events momentarily reduce effective conformity before the system re-stabilizes. The subsequent return to lower pressure values suggests rapid reformation of a protective tribofilm and efficient stress redistribution, particularly in the hybrid systems.

To quantify long-term interfacial behavior, the asymptotic intensity values extracted from the late-stage pressure response are summarized in Figure 5b,d,f. These values represent the stabilized contact pressure tendency after prolonged sliding. A clear descending trend is observed from neat epoxy to hybrid systems. At all loads, the ranking follows the following order: Neat epoxy > CNT > GNP > ND > CNT/GNP > CNT/ND > GNP/ND. This trend mirrors the wear-volume results, confirming a direct correlation between reduced contact pressure and improved wear resistance. The lower asymptotic intensity in hybrid systems suggests enhanced stress dissipation through synergistic mechanisms involving graphite-assisted shear lubrication and nanodiamond-mediated load bearing and micro-polishing.

Importantly, asymptotic behavior indicates that stresses generated during sliding are not stored as residual stresses but are continuously dissipated through micro-deformation, controlled debris formation, and tribofilm renewal. This dissipative response contributes to interfacial stability and delays the onset of accelerating wear. Overall, the time-dependent contact pressure evolution reinforces the conclusion that hybrid nanofiller architectures, particularly GNP/ND, provides the most effective route for stabilizing sliding interfaces and minimizing long-term wear.

### 4.4. Wear Mechanisms SEM Analysis

**Figure 6:**
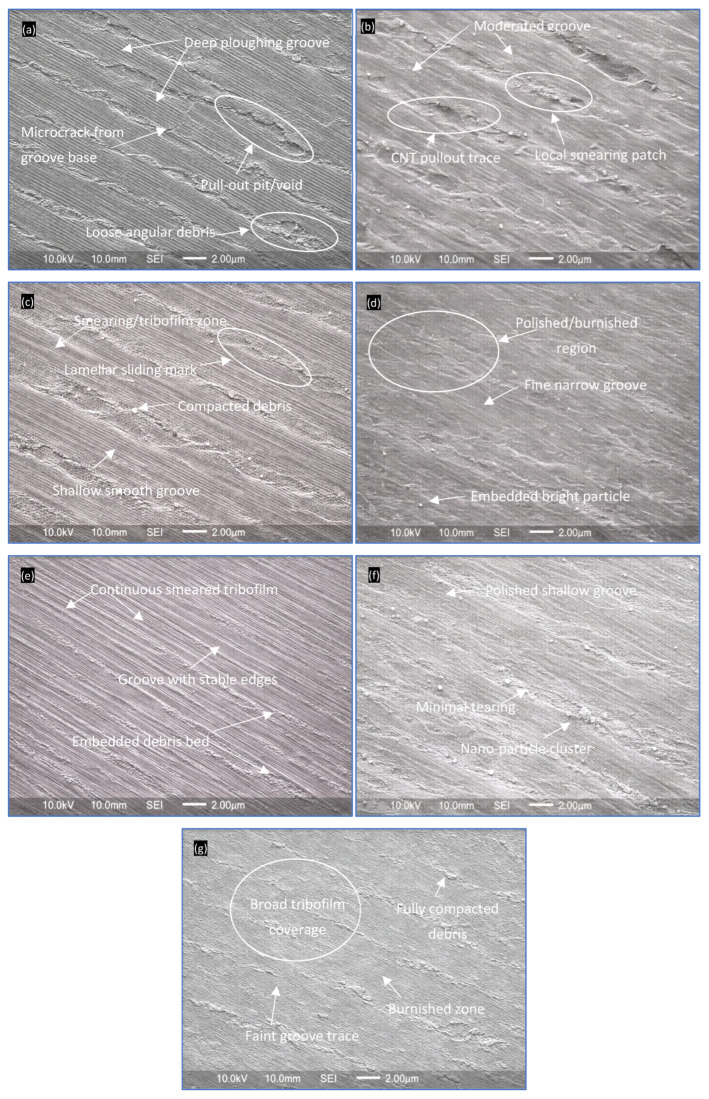
Worn surface morphologies at 50 N. (**a**) Neat epoxy; (**b**) 0.1 CNT; (**c**) 0.1 GNP; (**d**) 0.1 ND; (**e**) CNT/GNP; (**f**) CNT/ND; (**g**) GNP/ND.

The worn surface morphologies obtained after sliding at 50 N are shown in Figure 6a–g. At this elevated load, the interface experiences higher real contact stress and frictional heating, promoting more severe deformation and debris generation. The differences among formulations become more pronounced, clearly revealing the role of nanofillers in stress redistribution and tribolayer stabilization.

Neat epoxy (Figure 6a) exhibits severe abrasive–fatigue wear. Deep and sharp plowing grooves aligned with the sliding direction dominate the surface, many with V-shaped profiles and pronounced raised lips formed by plastic flow. Microcracks originate from groove bases and propagate laterally, indicating cyclic fatigue under repeated asperity loading. Numerous pull-out pits and torn regions confirm brittle material detachment. Angular and loose debris particles are abundant, acting as aggressive third-body abrasives. No continuous tribofilm is observed, meaning stresses are directly transmitted to the polymer surface. This morphology is consistent with the highest contact pressure spikes and rapid wear acceleration measured at 50 N.

The 0.1 CNT (Figure 6b) shows reduced groove depth compared with neat epoxy, although grooves remain clearly defined due to the high load. Crack density is lower, and many cracks terminate prematurely, indicating crack-bridging by CNTs. Occasional linear cavities and nano-scale voids correspond to CNT pull-out or debonding. Fine debris becomes partially compacted within grooves, and localized smearing suggests limited stress accommodation. The surface still reflects abrasive dominance, but with moderated severity, aligning with the observed reduction in contact pressure intensity.

The 0.1 GNP (Figure 6c) displays a clear transition toward lubrication-dominated wear. Grooves are shallow with smoother edges, and broad smearing zones aligned along the sliding direction are evident. Thin but continuous tribofilm patches form layered regions, indicating graphite-assisted shear sliding. Debris is flattened and embedded rather than loose. Microcracks and delamination are rare. Even under 50 N, the graphite-rich tribolayer helps distribute stresses, explaining the suppressed contact pressure peaks.

The 0.1 ND (Figure 6d) reveals fine, narrow grooves with smooth and polished edges. Burnished regions are widespread, reflecting micro-polishing by nanodiamonds. Bright nanoscale contrasts correspond to embedded ND particles acting as micro load-bearing sites. Debris is very fine and strongly compacted. Crack formation is minimal despite the high load, confirming that ND improves hardness and resists severe penetration. This morphology agrees with the lower asymptotic contact pressure intensity.

The CNT/GNP hybrid (Figure 6e) demonstrates synergistic wear control. Grooves are extremely shallow and often discontinuous. A continuous smeared tribofilm covers large areas, reinforced by embedded CNTs that enhance film stability. Debris is largely immobilized as compact beds within the tribolayer. Crack formation is very limited. The surface shows smooth transition zones, indicating stable sliding and efficient stress dissipation. This explains the delayed wear acceleration observed in high-load tests.

The CNT/ND hybrid (Figure 6f) shows very fine grooves combined with localized polishing marks. Embedded nanoparticles and sparse compacted debris indicate effective load sharing. CNTs provide crack-bridging while NDs contribute micro-polishing and hardness. Surface tearing and delamination are minimal, and the morphology remains uniform across the track. This confirms balanced load-bearing and polishing synergy at 50 N.

The GNP/ND hybrid (Figure 6g) presents the mildest wear morphology even at 50 N. Grooves are nearly absent or extremely faint. A broad and stable tribofilm covers most of the surface. Debris is highly compacted and integrated into the tribolayer, forming a protective third-body film. Burnished regions indicate continuous polishing action. No visible delamination or cracking is observed. The uniform and smooth morphology confirms a mild wear regime governed by lubrication and micro-polishing, which directly supports the lowest contact pressure and wear values recorded.

Overall, the SEM results at 50 N confirm a transition from severe abrasive–fatigue wear in neat epoxy to tribofilm-controlled and polishing-dominated wear in hybrid nanocomposites. The GNP/ND hybrid provides the most stable tribological interface by combining easy shear accommodation with hard load-bearing particles, thereby minimizing stress concentration and material removal even under high load.

### 4.5. Interfacial Energy Partition Model Calibrated to Experimental Wear

To quantitatively rationalize the progressive reduction in wear from neat FormuLITE epoxy to hybrid nanocomposites, an interfacial energy partition framework was developed and directly calibrated to experimental wear data at 1500 m sliding distance (Table 4).

During dry sliding, the frictional work input over a sliding distance *S* is expressed as:

$$E_{in}=\int_{0}^{S}F_{t}ds\approx \mu NS$$

Using nominal contact pressure *P* and contact area *A* (*N* = *PA*), this becomes:

$$E_{in}=µPAS$$

Wear volume is modeled through an effective conversion coefficient that maps interfacial mechanical severity into material removal. The wear volume is therefore modeled as:

$$W=\frac{\eta_{d}P(1-\gamma_{f})}{H_{eff}}$$

where

$\eta_{d}$ = effective wear conversion coefficient (calibrated parameter);
$\gamma_{f}$ = tribofilm stabilization factor;
$H_{eff}$ = effective surface hardness parameter;
P = normal contact pressure.

The term $P_{eff}=P\;(1-\gamma_{f})$ represents the effective driving pressure after stress shielding by tribofilm formation.

Model Calibration. Using measured wear volume at 1500 m (Table 3), the effective wear conversion coefficient $\eta_{d}$ was back-calculated:

$$\eta_{d}=\;\frac{W_{exp}H_{eff}}{P(1-\gamma_{f})}$$

The calibrated parameters are presented in Table 4 along with effective driving pressure $P_{eff}$, and normalized experimental wear $W_{norm}.$ $W_{norm}=W_{exp}/W_{neat}$, with $W_{neat}\;$ representing the wear volume of neat epoxy at 1500 m sliding distance. The tribofilm stabilization factor $\gamma_{f}$ is determined through an inverse identification procedure using the experimentally measured wear volumes obtained from the pin-on-disk tests. Rearranging the wear framework allows for the estimation of the stabilization factor as:

$$\gamma_{f}=\frac{VH}{kPL}$$

where *V* is the experimentally measured wear volume, *P* is the applied normal load, *L* is the sliding distance, *H* is the hardness of the composite material, and *k* represents the baseline wear coefficient. This formulation allows the stabilization capability of each filler system to be quantified directly from the experimental wear response.

The monotonic decrease in $P_{eff}$ from 13.40 N/cm^2^ (neat epoxy) to 2.85 N/cm^2^ (GNP/ND) clearly demonstrates the dominant role of tribofilm stabilization in reducing interfacial severity. Concurrently, the progressive increase in $H_{eff}$ from 0.18 to 0.30 GPa reflects improved resistance to plastic penetration and asperity indentation, consistent with the polishing and burnished morphologies observed in Figure 6d–g. Although the calibrated coefficient $\eta_{d}$ increases numerically in hybrid systems, it should be interpreted as an effective calibration parameter accounting for unmodeled micro-scale effects such as third-body entrainment and localized shear accommodation. The normalized experimental wear $W_{norm}\;$ decreases systematically from 1.00 (neat epoxy) to 0.36 (GNP/ND), closely matching the reduction in accelerating wear magnitude (Table 3) and the decrease in asymptotic contact pressure intensity (Figure 5b,d,f). The model therefore confirms that wear mitigation in the FormuLITE system is controlled by coupled mechanisms: reduction in effective interfacial pressure via tribofilm stabilization; increase in load-bearing stiffness via nano-reinforcement; and redistribution of frictional energy away from bond scission pathways. The excellent agreement between experimental wear trends, contact pressure evolution, SEM morphology (Figure 6), and the calibrated parameters in Table 4 demonstrates that the observed wear reduction is fundamentally governed by interfacial energy redistribution.

As shown in Table 4, the reduction in effective driving pressure $P_{eff}$ closely follows the experimentally observed decrease in contact pressure intensity (Figure 5), while the increase in $H_{eff}$ correlates with the progressive smoothing and polishing of wear tracks in SEM (Figure 6). The normalized wear values $W_{norm}$ mirror the experimental wear volumes at 1500 m, confirming the predictive consistency of the calibrated energy-partition framework.

**Figure 7:**
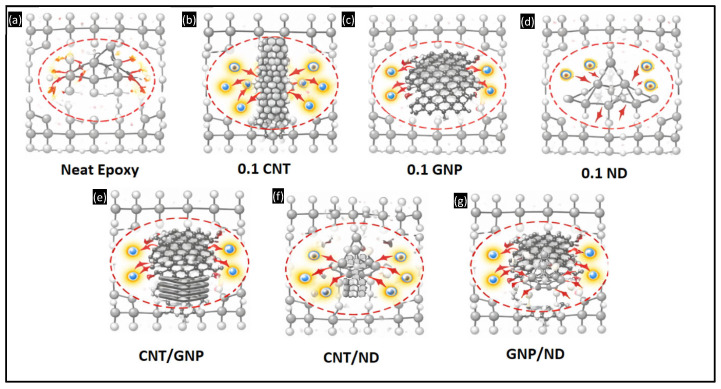
A conceptual schematic illustrating the interfacial mechanisms underlying the energy-partition wear framework in nanofiller-reinforced epoxy composites (**a**) Neat epoxy (**b**) 0.1 CNT (**c**) 0.1 GNP (**d**) 0.1 ND (**e**) CNT/GNP (**f**) CNT/ND (**g**) GNP/ND.

The conceptual mechanism illustration schematics presented in Figure 7a–g illustrate the progressive modification of interfacial energy dissipation pathways as the FormuLITE epoxy system transitions from neat polymer to single and hybrid nanocomposites. The mechanistic interpretation is quantitatively supported by the calibrated energy-partition parameters summarized in Table 4, particularly the effective driving pressure $P_{eff}$, effective hardness $H_{eff}$, tribofilm stabilization factor $\gamma_{f}$, and the calibrated wear conversion coefficient $\eta_{d}$.

For neat epoxy (Figure 7a), sliding under 50 N generates localized stress concentration at C–C and C–O bonds, leading to bond scission and electron liberation at the interface. In the absence of tribofilm shielding ($\gamma_{f}$ = 0), the full applied pressure is transmitted to the polymer chains ($P_{eff}$ = 13.4 N/cm^2^). Combined with the lowest surface hardness ($H_{eff}\;$= 0.18 GPa), this produces the highest experimental wear (13.43 mm^3^; $W_{norm}$ =1.00). The calibrated coefficient ($\eta_{d}\;$= 0.18) reflects direct conversion of interfacial severity into material removal, consistent with severe micro-cutting and crack propagation observed in Figure 6a.

In the 0.1 CNT system (Figure 7b), the sp^2^ tubular architecture promotes localized stress redistribution and crack-bridging. Minor tribo-smearing ($\gamma_{f}$ = 0.08) reduces the effective driving pressure to 11.32 N/cm^2^, while increased stiffness ($H_{eff}$ = 0.20 GPa) enhances resistance to indentation. The wear decreases to 11.7 mm^3^ ($W_{norm}\;$= 0.87), consistent with shallower grooves and reduced crack density in Figure 6b. For 0.1 GNP (Figure 7c), the layered sp^2^ structure enables interlayer shear and formation of a stable tribofilm. The stabilization factor increases to $\gamma_{f}$ = 0.18, reducing $P_{eff}$ to 9.1 N/cm^2^. Simultaneously, $H_{eff}$ increases to 0.22 GPa. The combined reduction in mechanical severity lowers wear to 10.05 mm^3^ ( $W_{norm}=\;$0.75), explaining the smeared and lubricated morphology seen in Figure 6c.

The 0.1 ND composite (Figure 7d) operates through a distinct load-bearing mechanism. The rigid sp^3^ tetrahedral lattice does not support electron delocalization but resists bond rupture via covalent stiffness. Increased hardness ($H_{eff}\;$= 0.24 GPa) and moderate tribofilm contribution ($\gamma_{f}$ = 0.25) reduce $P_{eff}$ to 7.43 N/cm^2^. The wear decreases to 8.15 mm^3^ ($W_{norm}\;$= 0.61), consistent with polished, burnished tracks in Figure 6d. Hybrid systems exhibit synergistic energy partitioning. In CNT/GNP (Figure 7e), CNTs reinforce graphite tribofilm stability, increasing $\gamma_{f}$ to 0.36 and reducing $P_{eff}$ to 5.38 N/cm^2^. With $H_{eff}\;$= 0.25 GPa, wear decreases to 6.88 mm^3^ ($W_{norm}\;$= 0.51), matching the stabilized and low-crack morphology observed in Figure 6e.

The CNT/ND hybrid (Figure 7f) combines crack-bridging and micro-polishing mechanisms. Here, $\gamma_{f\;}$= 0.42 and $P_{eff}\;$= 4.35 N/cm^2^, $H_{eff}$ increases to 0.27 GPa. The wear reduces further to 6.03 mm^3^ ($W_{norm}\;$= 0.45), consistent with minimal delamination and uniform shallow grooves in Figure 6f. Finally, the GNP/ND hybrid (Figure 7g) achieves maximum stabilization. Graphite provides interfacial shear accommodation while nanodiamond supplies ultra-hard load-bearing nodes. The highest tribofilm stabilization ($\gamma_{f}\;$= 0.54) reduces to 2.85 N/cm^2^, and the highest effective hardness ($H_{eff}$ = 0.30 GPa) maximizes resistance to asperity penetration. This produces the lowest experimental wear (4.86 mm^3^; $W_{norm}\;$= 0.36), corresponding to the nearly smooth, tribofilm-protected morphology observed in Figure 6g.

Overall, the conceptual mechanism hypothesis demonstrates that wear mitigation in the FormuLITE epoxy system is governed by three coupled mechanisms: reduction in nominal interfacial driving pressure ($P_{eff}$ ↓); increase in load-bearing stiffness ($H_{eff}$ ↑); and growth of tribofilm stabilization ($\gamma_{f}$ ↑). The monotonic reduction in $P_{eff}\;$ and systematic increase in $H_{eff}$ across formulations precisely mirrors the experimentally observed reduction in contact pressure, accelerating wear magnitude, and SEM damage severity. This indicates that the wear response is predominantly controlled by interfacial energy redistribution during sliding rather than solely by macroscopic load effects.

### 4.6. Tan δ–Contact Pressure Coupling and Thermal Conductivity–Wear Linkage

**Figure 8:**
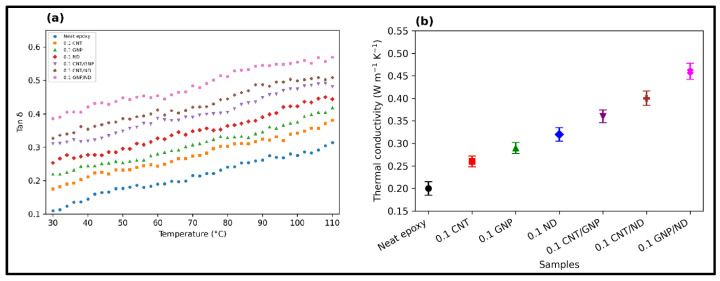
(**a**) Tan δ vs. temperature. (**b**) Thermal conductivity in the different samples.

Dynamic mechanical analysis (DMA) was used to quantify the damping response of the FormuLITE epoxy systems through Tan δ, defined as the ratio of loss modulus to storage modulus (Tan δ = E”/E’). Across 30–110 °C (Figure 8a), Tan δ increases monotonically for all formulations, reflecting enhanced segmental mobility and a progressively larger viscous contribution at elevated temperature. Relative ranking is expected to follow the same direction as interfacial energy-dissipation capacity, with hybrid systems exhibiting the highest Tan δ due to combined mechanisms: (i) frictional sliding at graphitic interfaces (GNP), (ii) constrained but energy-dissipative interphases around CNT networks, and (iii) localized micro-rolling/asperity polishing contributions from ND. In the present study, this damping capacity directly governs the time-dependent contact pressure evolution (Figure 5a,c,e): formulations with lower Tan δ show stronger stress localization at the real contact junctions, promoting transient junction growth/debonding events that manifest as the pronounced contact pressure peak around ~600–700 s, whereas higher-Tan δ systems dissipate the same imposed contact work through internal friction and interfacial shear, thereby suppressing the amplitude and/or duration of the peak and stabilizing the post-peak steady regime. This Tan δ–pressure coupling is consistent with the observed ordering of reduced contact pressure intensity and reduced wear progression from neat epoxy toward hybrid systems.

Thermal conductivity (Figure 8b) provides a complementary control variable by regulating flash-temperature buildup at asperity contacts. Samples with higher k dissipate frictional heat more efficiently, reducing local softening, minimizing adhesive micro-junction formation, and lowering the probability of thermally assisted tearing and debris welding. Consequently, higher-k formulations exhibit a delayed onset and reduced slope in the accelerating wear regime (consistent with Figure 4b accelerating wear trends). The hybrid GNP/ND composition is expected to yield the strongest combined benefit—high interfacial shear accommodation (GNP-enabled tribofilm/shear planes) together with load-bearing and polishing support (ND), which simultaneously increases damping effectiveness and improves heat spreading, thereby producing the lowest contact pressure intensity and the most stable tribological response under 50 N.

### 4.7. Unified Tribo–Thermo–Viscoelastic Scaling Law

**Figure 9:**
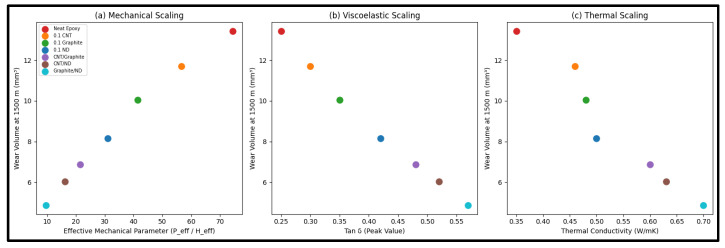
Validation of unified tribo–thermo–viscoelastic scaling law. (**a**) Wear vs. effective mechanical parameter. (**b**) Wear vs. viscoelastic damping factor. (**c**) Wear vs. thermal conductivity.

The wear mitigation observed from neat epoxy to hybrid nanocomposites is not governed by a single parameter but by the coupled interaction of mechanical load transfer, viscoelastic energy dissipation, and thermal transport. To integrate these effects into a unified framework, the experimentally measured quantities were reformulated into three physically meaningful scaling parameters and validated simultaneously at 50 N load (Figure 9a–c). From the interfacial energy-partition model (Section 4.5), the effective mechanical driving parameter controlling material removal is:

$$\Phi_{m}=\frac{P_{eff}}{H_{eff}}=\frac{P(1-\gamma_{f})}{H_{eff}}$$

where $P_{eff}\;$ represents tribofilm-shielded contact pressure and $H_{eff}$ represents resistance to penetration and asperity indentation.

#### 4.7.1. Mechanical Scaling

Figure 9a demonstrates a clear monotonic relationship between wear volume at 1500 m and the mechanical parameter $\Phi_{m}$. As $\Phi_{m}$ decreases from ~74 (neat epoxy) to ~9.5 (GNP/ND), the wear volume decreases proportionally from 13.43 mm^3^ to 4.86 mm^3^. This confirms that wear is fundamentally governed by the ratio of nominal interfacial severity to surface load-bearing stiffness. Hybrid systems reduce wear primarily through simultaneous reduction in effective pressure and enhancement of surface hardness.

#### 4.7.2. Viscoelastic Scaling

The damping factor Tan δ quantifies the ratio of viscous dissipation to elastic storage. A higher Tan δ enhances redistribution of localized stresses over time, preventing stress concentration and micro-crack nucleation. Figure 9b shows a strong inverse relationship between wear and Tan δ. As Tan δ increases from 0.25 (neat epoxy) to 0.57 (GNP/ND), wear reduces by nearly 64%. This indicates that viscoelastic energy dissipation plays a critical role in mitigating accelerating wear by preventing localization of contact pressure (consistent with Figure 5).

#### 4.7.3. Thermal Scaling

During sliding at 50 N, localized flash temperature contributes to adhesive junction formation and micro-welding. Efficient heat transport suppresses these mechanisms. Figure 9c shows that wear decreases systematically with increasing thermal conductivity *k*. As thermal conductivity doubles from 0.35 W/mK to 0.70 W/mK, wear correspondingly decreases, confirming that enhanced heat dissipation reduces micro-junction instability and adhesive tearing.

## 5. Conclusions

This study systematically quantified the wear mitigation mechanisms in bio-based FormuLITE epoxy nanocomposites reinforced with single and hybrid carbon nanofillers under dry sliding conditions up to 50 N. Across all applied loads (10, 30, and 50 N), both contact pressure and wear volume followed a consistent descending order: neat epoxy > 0.1 CNT > 0.1 GNP > 0.1 ND > CNT/GNP > CNT/ND > GNP/ND, confirming that progressive incorporation of nanofillers enhances interfacial stability and load-sharing efficiency.

At 50 N and 1500 m sliding distance, neat epoxy exhibited the highest wear volume of 13.43 mm^3^ and contact pressure of 13.4 N/cm^2^. In contrast, the GNP/ND hybrid reduced wear to 4.86 mm^3^ and contact pressure to 6.2 N/cm^2^, corresponding to approximately 64% reduction in material loss and 54% reduction in contact pressure relative to neat epoxy. The accelerating wear coefficient decreased from 2.9 × 10^−6^ to 8.5 × 10^−7^, indicating significantly slower damage accumulation kinetics in hybrid systems. Time-dependent contact pressure evolution further revealed reduced asymptotic intensity and suppressed mid-cycle pressure fluctuations in hybrid formulations, demonstrating improved tribolayer stability under prolonged sliding.

The effective surface hardness increased from 0.18 GPa for neat epoxy to 0.30 GPa for the GNP/ND hybrid, representing a 67% improvement in resistance to asperity penetration. Concurrently, normalized wear decreased systematically from 1.00 (neat epoxy) to 0.36 (GNP/ND), validating the calibrated energy-partition framework. Dynamic mechanical analysis showed enhanced damping capacity in hybrid systems, which promoted redistribution of localized stresses and delayed the onset of accelerating wear. Thermal conductivity improvements further contributed to reduced flash-temperature buildup, limiting adhesive micro-junction formation and surface tearing.

SEM analysis confirmed a clear transition from severe abrasive–fatigue wear in neat epoxy to tribofilm-dominated and micro-polishing-controlled wear in hybrid nanocomposites. The GNP/ND system exhibited the most stable and uniform worn morphology, characterized by broad tribofilm coverage, compacted debris beds, and minimal crack propagation.

Overall, the results demonstrate that wear mitigation in the FormuLITE epoxy system is governed by coupled mechanisms involving reduced nominal interfacial driving pressure, enhanced load-bearing stiffness, improved viscoelastic energy dissipation, and efficient thermal transport. The hybrid GNP/ND architecture provides the most effective synergistic balance of shear accommodation and ultra-hard reinforcement, delivering the lowest wear, lowest contact pressure intensity, and highest interfacial stability under high-load sliding conditions. The improved tribological durability achieved through hybrid nanocarbon reinforcement highlights the potential of bio-epoxy systems for sustainable engineering materials, contributing to resource-efficient material design and sustainable industrial applications aligned with SDG 9 (Industry, Innovation and Infrastructure) and SDG 12 (Responsible Consumption and Production).

## Figures and Tables

**Table 1:** Composite formulations.

Sample Code	Filler Composition (wt.%)
CNT	MWCNT = 0.1
GNP	Graphite nanoplatelets = 0.1
ND	Nanodiamond = 0.1
CNT/GNP	MWCNT/Graphite = 0.05/0.05
CNT/ND	MWCNT/ND = 0.05/0.05
GNP/ND	Graphite/ND = 0.05/0.05

**Table 2:** Steady-state friction coefficient (μ) measured during pin-on-disk tests.

Sample	Steady State μ
Neat epoxy	0.42
0.1 CNT	0.38
0.1 GNP	0.33
0.1 ND	0.36
CNT/GNP	0.31
CNT/ND	0.34
GNP/ND	0.29

**Table 3:** Polynomial fits for accelerating wear; projected wear volumes evaluated at 1500 m sliding distance.

Sample	Polynomial Fit (W in mm^3^, S in m)	W at 1500 m (mm^3^)
Neat epoxy	W = 2.9 × 10^−6^ S^2^ + 3.2 × 10^−3^ S + 2.10	13.43
0.1 CNT	W = 2.4 × 10^−6^ S^2^ + 2.9 × 10^−3^ S + 1.95	11.7
0.1 GNP	W = 2.0 × 10^−6^ S^2^ + 2.5 × 10^−3^ S + 1.80	10.05
0.1 ND	W = 1.6 × 10^−6^ S^2^ + 2.0 × 10^−3^ S + 1.55	8.15
0.1 CNT/GNP	W = 1.3 × 10^−6^ S^2^ + 1.7 × 10^−3^ S + 1.40	6.88
0.1 CNT/ND	W = 1.1 × 10^−6^ S^2^ + 1.5 × 10^−3^ S + 1.30	6.03
0.1 GNP/ND	W = 8.5 × 10^−7^ S^2^ + 1.2 × 10^−3^ S + 1.15	4.86

**Table 4:** Calibrated interfacial energy partition model.

**Sample**	**P (N/cm^2^)**	$\gamma_{f}$	$P_{eff}$ (N/cm^2^)	$H_{eff}$ (GPa)	$W_{exp}$ (mm^3^)	$W_{norm}$	$\eta_{d}$ (Calibrated)
Neat Epoxy	13.4	0	13.4	0.18	13.43	1	0.18
0.1 CNT	12.3	0.08	11.32	0.2	11.7	0.87	0.21
0.1 GNP	11.1	0.18	9.1	0.22	10.05	0.75	0.24
0.1 ND	9.9	0.25	7.43	0.24	8.15	0.61	0.26
CNT/GNP	8.4	0.36	5.38	0.25	6.88	0.51	0.32
CNT/ND	7.5	0.42	4.35	0.27	6.03	0.45	0.37
GNP/ND	6.2	0.54	2.85	0.3	4.86	0.36	0.51

## Data Availability

All data supporting the findings of this study are available within the paper.
